# Adolescents’ perspectives on socio-cultural influences on overweight and obesity in African countries: a systematic scoping review of qualitative studies

**DOI:** 10.1007/s13679-026-00767-8

**Published:** 2026-09-30

**Authors:** Emmanuel Osei Bonsu, Steven Allender, Erik Martin, Richmond Aryeetey, Colin Bell

**Affiliations:** 1 Deakin Centre for Global Preventative Health and Nutrition, Faculty of Health, Deakin Institute for Health Transformation, Deakin University, Geelong, Australia; 2 School of Medicine, Faculty of Health, Deakin University, Geelong, Australia; 3 Department of Population, Family and Reproductive Health, University of Ghana, Legon, Ghana

**Keywords:** Obesity, Overweight, Socio-cultural influences, Adolescent

## Abstract

**Introduction:**

Adolescent obesity is a global public health concern, with increasing prevalence rates in many African countries. Studies have highlighted diverse social and cultural influences on weight, but there is uncertainty about how adolescents in Africa perceive and experience these influences. This review aims to describe adolescents’ perspectives on how cultural beliefs and norms, peers, family, and media influence weight-related behaviours, body weight, and experiences of weight stigma in African countries.

**Methods:**

We systematically searched Scopus, PubMed, Embase, Web of Science, CINAHL, PsycINFO and SocINDEX for qualitative studies on socio-cultural determinants of adolescents’ overweight and obesity in African countries. Study quality was assessed using the Critical Appraisal Skills Programme (CASP) for qualitative research. Using a narrative synthesis approach guided by socio-cultural theory, we summarised and interpreted findings across studies to describe adolescents’ perspectives on how cultural beliefs, peers, family, and media influence weight and experiences of weight stigma.

**Results:**

Of 1,410 articles identified, 17 met the inclusion criteria from eight African countries. Eight studies were conducted in South Africa, while the remaining studies were conducted in Benin, Botswana, Cameroon, Ethiopia, Gambia, Kenya, and Uganda, with no eligible studies identified from Northern or Central Africa. Synthesis of these studies revealed that adolescents perceived peers and parents/families as important influences on their food choices, in the direction of unhealthy options, and on their physical activity levels. Adolescents also described media as shaping these behaviours and perceptions of body image. Several studies reported that overweight or obesity was associated with wealth and health, while thinness was associated with disease or illness. Weight stigma was reported among adolescents irrespective of their weight status.

**Conclusion:**

Adolescents from eight African countries perceived peers, family members, and media as important socio-cultural influences on their weight-related behaviours, perceptions of body weight, and experiences of weight stigma. Interventions to prevent and manage adolescent obesity may be more effective and better received if these perceived socio-cultural influences are integrated into the design.

**Supplementary Information:**

The online version contains supplementary material available at https://doi.org/10.1007/s13679-026-00767-8.

## Introduction

Adolescent overweight and obesity are global public health concerns [1]. Rapid urbanisation, economic growth, and changing dietary patterns are driving population-level nutrition transition, contributing to increasing rates of unhealthy weight gain in all countries, including those in Africa [2]. Countries in Africa have seen nutrition-related diseases emerge at a faster rate and at a lower economic level than industrialised countries [3]. Recent evidence from sub-Saharan African countries indicates a growing burden of adolescent overweight and obesity, with reported prevalence estimates ranging from 4.1% to 26% for overweight and from 0.2% to 13.5% for obesity [4].

Cultural values and societal norms are known to influence what constitutes a desirable body shape and size [5, 6]. In many African countries for example, large body size is perceived to be associated with prosperity, healthiness, and high social status [7–9]. Dietary practices and physical activity patterns (for example, increased opportunities for physical activity at school for boys compared to girls), often established in childhood, affect adolescents’ energy balance and contribute to overweight and obesity [10, 11]. Adolescence is a period in life marked by significant changes in brain development, body composition, behavioural changes, physical growth, psychological health, and socio-cultural influences [12]. Living with overweight or obesity during this stage of life can impact quality of life, as it raises the risk of heart disease, mental health issues, social difficulties, diabetes, cancer, stroke, obesity in adulthood, and even premature death [13, 14]. In adolescence, the brain regions responsible for decision-making are still developing [15] making adolescents more susceptible than any other age group to socio-cultural influences that shape perceptions of body weight [16]. Adolescents share environments with their peers and families, making their behaviours and lifestyles easy to imitate [17, 18]. If these behaviours are sedentary or lead to unhealthy food consumption, they are likely to result in unhealthy weight gain [19, 20]. For some adolescents, they can also lead to disordered eating and related health problems, beyond the scope of this review [21].

Potentially exacerbating socio-cultural influences on adolescent weight are media portrayals of idealised body standards, along with frequent advertising of unhealthy foods and sedentary behaviours such as gaming [22, 23]. Increased exposure to social media appears to be increasing weight stigma experiences for adolescents, which includes stereotyping, labelling, or being subjected to discrimination from peers, families, and the media based on their weight[24, 25]. These stigmatising behaviours are rooted in the culturally and socially constructed ideals and expectations mentioned earlier[24, 26] and can negatively impact adolescents’ mental health and self-esteem [27–29].

Several reviews have examined determinants of overweight and obesity in Africa; however, socio-cultural influences are rarely explored. One review for example, focused on black women in South Africa and environmental factors influencing unhealthy weight [5]. Similarly, a systematic review of obesity determinants in West Africa synthesised demographic, lifestyle, biological, and socioeconomic risk factors but did not specifically explore adolescents’ perceptions of socio-cultural influences [30]. A further review conducted in Sub-Saharan Africa focused broadly on factors contributing to overweight and obesity among adult women [31].

To our knowledge, no previous review has synthesised qualitative evidence on how African adolescents perceive the influence of peers, family, and media on overweight and obesity. Quantitative studies can provide evidence on associations between socio-cultural factors and adolescent weight-related outcomes but generally provide limited insight into how adolescents perceive and experience these factors within their social and cultural contexts. Therefore, this review aims to synthesise existing peer-reviewed literature and describe adolescents’ perspectives on the socio-cultural influences on overweight and obesity in African countries, specifically their perceptions of how cultural beliefs and norms, peers, family, and media influence adolescents’ weight and experiences of weight stigma.

## Methods

### Study Design

We conducted a systematic scoping review following the Preferred Reporting Items for Systematic and Meta-Analysis Scoping Review (PRISMA-ScR) guidelines [32]. The PRISMA framework provides a standardised and systematic method for reviewing published evidence. We have registered this protocol with the Open Science Framework (OSF) with registration doi: https://doi.org/10.17605/OSF.IO/59MAB.

### Search Strategy

A comprehensive set of search terms was developed to search Scopus, PubMed, Embase, Web of Science, CINAHL, PsycINFO, SocINDEX, representing the main databases on health research. A supplementary search was conducted in Google Scholar, African Index Medicus and Africa Journal Online for socio-cultural determinants literature that may not be found in the health research databases. The search was initially conducted on 28th April 2025 and updated on 29th May 2026. Using the PEO (Population, Exposure, Outcome) framework, specific search terms and keywords were defined using population of interest, the exposure of interest, study design, and outcome of interest (unhealthy weight, weight stigma), and concepts and search terms were identified in consultation with Deakin University library staff (Table 1). Related terms and synonyms within each search concept were combined using the Boolean operator “OR”, while the search concepts were combined using “AND”. Both free-text keywords and relevant controlled vocabulary were used in the search strategy. In PubMed, relevant Medical Subject Headings (MeSH) were used alongside free-text terms, with equivalent subject headings adapted to the indexing systems of other databases where applicable. We also conducted backward citation tracking by screening the reference lists of included studies and forward citation tracking by identifying studies that cited the included articles on Google Scholar.

**Table 1:** Search strategy

#	Concept	Search terms	Subject headings/Controlled vocabulary (e.g. MeSH)
Exposure	Social-cultural determinants	socio-cultural OR "cultural norm*” OR “Social Values” OR “cultural value*” OR "social expectation*” OR “Peer Influence" OR “peer pressure*” OR family OR “family influence*” OR “family environment” OR “Social Media” OR “media influence” OR “social pressure” OR “peer environment” OR determinant*	Peer influence Social Values Social Media Family
		AND	
Population	Adolescent	Adolescent OR Adolescent* OR Teenage* OR Teen* OR Youth* OR “Young adult” OR “Young people”	Adolescent
		AND	
Outcome	Overweight/obesity	Obesity OR Overweight OR obes* OR overweight* OR “Body Mass Index” OR “Body Weight” OR “Body Mass Index” OR BMI OR “body weight” OR Adipos* OR Adiposity OR “excess weight” OR “excessive weight” OR “unhealthy weight*” OR Fat OR “Fat accumulation” OR “weight bias” OR “weight discriminat*” OR “weight stigma” OR “Weight Prejudice”	Obesity Overweight Body Mass Index Body weight Weight Prejudice
		AND	
Population	Africa	Africa OR Algeria OR Angola OR Benin OR Botswana OR “Burkina Faso” OR Burundi OR “Cabo Verde” OR Cameroon OR “Central African Republic” OR Chad OR Comoros OR Congo OR “Democratic Republic of Congo” OR “Cote d’Ivoire” OR Djibouti* OR Egypt* OR “Equatorial Guinea*” OR Eritrea* OR Eswatini* OR Ethiopia* OR Gabon* OR Gambia* OR Ghana* OR Guinea* OR Guinea-Bissau* OR Kenya* OR Lesotho* OR Liberia* OR Libya* OR Madagascar* OR Malawi* OR Mali* OR Mauritania* OR Mauritius* OR Morocco* OR Mozambique* OR Namibia* OR Niger* OR Nigeria* OR Rwanda* OR “Sao Tome and Principe*” OR Senegal* OR Seychelles* OR “Sierra Leone*” OR Somalia* OR “South Africa*” OR “South Sudan*” OR Sudan* OR Tanzania* OR Togo* OR Tunisia* OR Uganda* OR Zambia* OR Zimbabwe*	Africa
		AND	
Outcome	Qualitative	“Qualitative Research” OR “Focus Groups” OR “qualitative stud*” OR qualitative* OR narrative* OR descriptive* OR exploratory* OR perception* OR view* OR attitude OR “thematic analysis” OR “content analysis” OR phenomenological OR ethnographic OR “grounded theory” OR “case study” OR “narrative inquiry” OR “participant observation” OR “group model*”	Qualitative Research Focus group

### Researcher Positionality and Reflexivity

The review team comprised two authors from Ghana and three authors from Australia. The Ghanaian authors brought contextual familiarity with African social and cultural settings. Australian authors brought expertise in public health and obesity-related research. Authors shared the view that prevention of obesity and weight-related stigma is possible and that this will improve adolescent health. To support reflexivity and minimise the influence of individual perspectives, data extraction and quality assessment were conducted independently, and interpretations were discussed among the review team until consensus was reached.

### Conceptual Framework

The theoretical framework adopted for the review was the socio-cultural theory, which theorises that individual learning and development are fundamentally shaped by social interactions, cultural norms and immediate environments [33]. Drawing on this theory, our underlying hypothesis is that social norms, cultural values, family, peer, and media, shape adolescent behaviours, influencing weight and experiences of weight stigma [5, 6, 34, 35]. Also, these perceived influences are modifiable and transferable to other locations and contexts [36]. Family, peer, and media influences were the initial areas of analytical focus. However, the framework was applied flexibly to identify other relevant influences reported by adolescents in the included studies. We used this framework to understand how adolescents perceived the influence of family, peers, and the media on their weight, or in other words, to understand adolescents’ own perspective on how these influences shape their learning and development.

### Inclusion and Exclusion Criteria

Studies were included if they: (1) assessed or described socio-cultural determinants of overweight/obesity. Our working definition of overweight and obesity was based on the World Health Organization age- and gender-specific definitions, but we included all studies that mentioned overweight or obesity, (2) involved adolescents between the ages of 10–19 years of ages, consistent with the World Health Organization’s definition of adolescence [37], (3) were conducted in African countries, (4) employed qualitative methodologies, and (5) were published in English in peer-reviewed journals. No restrictions were applied based on publication year. Studies were excluded if they used quantitative methods, focused on adults or populations outside the 10–19 years age range, or were conducted outside Africa. No additional exclusion criteria were applied to studies that met all inclusion criteria.

### Data Screening and Extraction

All articles were imported into Covidence software for screening. Three independent reviewers (EB, EM and RA) conducted the title and abstract screening. Inter-rater agreement among the three reviewers was estimated using Fleiss kappa (k = 0.79). Disagreements were resolved through discussion and consensus. This was followed by a full-text review, and articles were assessed based on the predefined inclusion criteria above by two independent researchers (EB, RA), with an inter-rater agreement of Cohen’s Kappa = 0.81. Discrepancies regarding the final list of included articles were resolved through discussion, ensuring that all researchers agreed with the final selection. Included articles were imported into Microsoft Excel for data extraction. Data extraction was conducted by two independent (EB, CB) reviewers using data extraction form. Following extraction, the data extracted by the two reviewers were compared, and any discrepancies were discussed and resolved through consensus. Extracted data from each study included authors, year of publication, country, study design, sample size, sampling technique, method of data collection, theoretical framework, data analysis, key findings and study limitations that were relevant to the study objectives. Any disagreements in data extraction were resolved through discussion and consensus between the two reviewers.

### Quality Assessment

We utilised multiple databases to minimise the risk of coverage bias and to capture a wide range of relevant studies. The Critical Appraisal skills Programme (CASP) for qualitative research was used for assessing the risk of bias [38]. The CASP tool is designed to rigorously assess the methodological quality of qualitative research to ensure that potential biases are identified. It comprises ten structured questions used to evaluate the relevance, validity, and reliability of qualitative studies. Each item was assessed as “Yes”, “No”, or “Can’t determine”. For scoring purposes, each criterion was assigned a score of 1 if it was met and 0 if it was not met or could not be determined, resulting in a total score ranging from 0 to 10. Higher scores indicated better methodological quality. Two independent reviewers (EB, CB) conducted the quality assessment of the included studies and differences in findings were resolved through discussion.

### Data Analysis and Synthesis

The extracted qualitative findings were analysed using deductive thematic analysis and narrative synthesis. Initial coding was guided by a coding framework informed by socio-cultural theory and the predefined review objectives (Appendix 1). The framework included themes related to peer, family, media, and cultural influences, as well as adolescents’ experiences of weight stigma, with corresponding codes and definitions to guide the categorisation of findings. The analysis was primarily deductive, guided by the coding framework, but additional codes and themes were developed inductively when relevant findings did not fit within the predefined categories. This approach allowed other relevant influences reported in the included studies to be captured. Relevant findings from each study were coded based on their meaning and content, and similar codes were compared and grouped into categories and themes. Coding and theme development were conducted by EB and reviewed by CB, EM, SA, and RA, with differences in coding or interpretation resolved through discussion and consensus.

A narrative synthesis was used to summarise and interpret the findings while preserving the contextual information reported in the included studies. Themes were compared across studies to identify similarities, differences, and recurring patterns. The narrative synthesis integrated these themes to provide a comprehensive understanding of adolescents’ perceived socio-cultural, economic, and environmental influences on weight-related behaviours, body weight, and experiences of weight stigma.

## Results

**Fig. 1:**
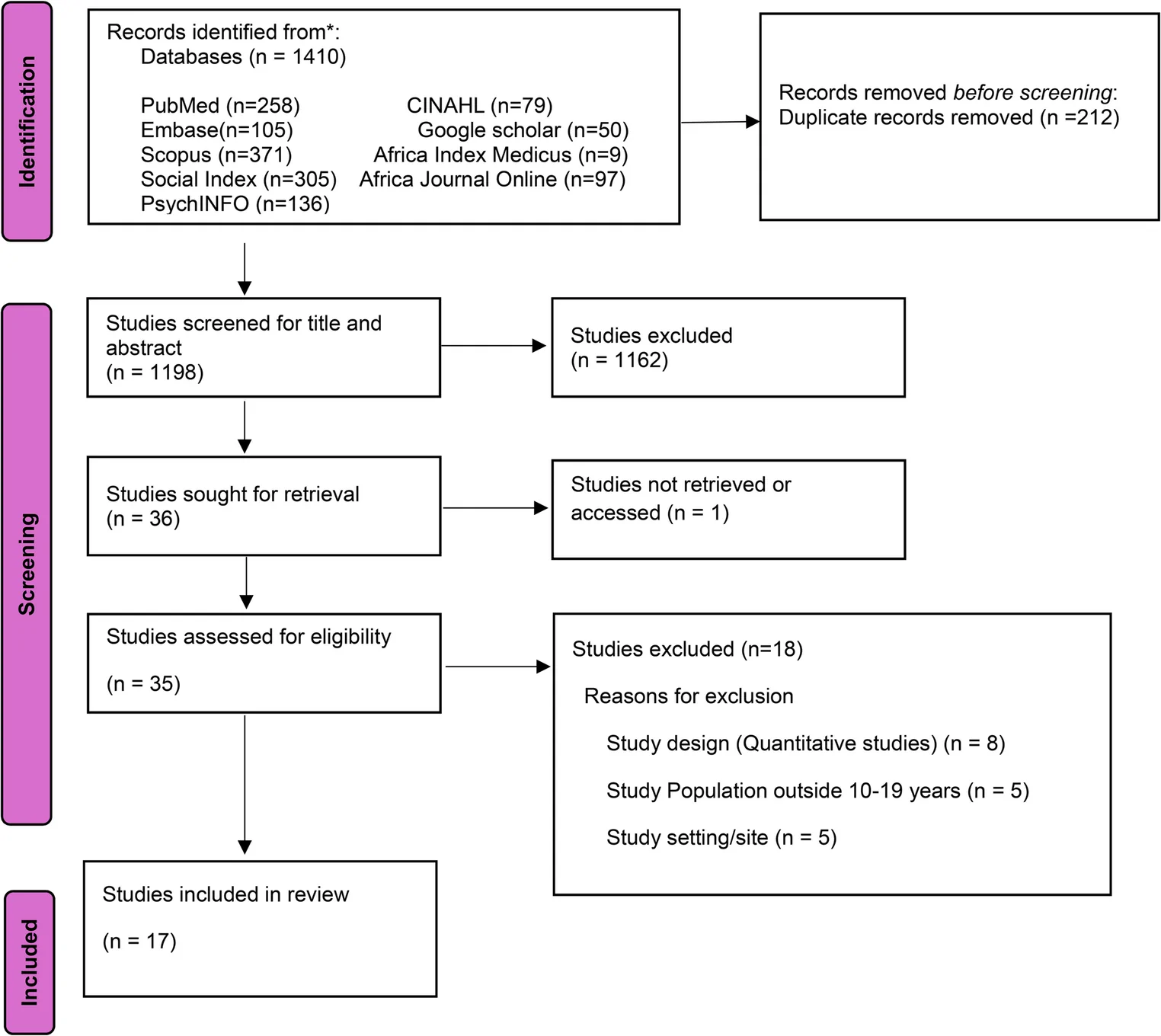
Prisma flow diagram

The search yielded 1,410 articles. There were 212 duplicate studies which were removed remaining 1,198 studies eligible for title and abstract screening. A total of 35 articles were eligible for full-text screening, and 18 studies were excluded because they employed a quantitative study design, were not conducted in Africa, or focused on children under five and adult populations (20 years of age and above). Seventeen of these studies met the inclusion criteria and had data extracted (Fig. 1).

A total of 590 adolescents participated in the 17 included studies. Out of 590 adolescents, 380 were females, and 81 were males. Several studies included both male and female adolescents but did not report their numbers separately by gender. Sample size varied, with the smallest study enrolling nine adolescents and the largest enrolling 102 adolescents. The included studies were conducted in urban (*n* = 3), rural (*n* = 4), or mixed urban-rural settings (*n* = 4), and six studies (*n* = 6) did not report the setting. Recruitment occurred through schools (*n* = 7), communities (*n* = 4), both schools and communities (*n* = 1), and a university (*n* = 1), with four studies not reporting the recruitment setting (*n* = 4). Almost half of the included studies (*n* = 8) were conducted in South Africa. The remaining studies were conducted in Gambia (*n* = 2), Ethiopia (*n* = 2), Botswana (*n* = 1), Kenya (*n* = 1), Benin (*n* = 1), Uganda (*n* = 1) and Cameroon (*n* = 1). There were no studies from Northern or Central Africa.

### Theoretical Frameworks and Data Collection Methods

Only six of the 17 included studies specified theoretical frameworks guiding their research. Four studies employed socio-ecological model [39–42], one used grounded theory[43] and another applied social cognitive theory [44]. These frameworks were used to guide study designs, data collection and analysis in understanding adolescents’ perceptions of social and cultural influences on their weight-related behaviours and body weight.

Eleven of the studies employed focus group discussions, six in-depth interviews and one group model building to gather data from adolescents. To analyse the data, fourteen of the studies employed thematic analysis, two studies used content analysis, and the group model building study produced a causal loop diagram (Table 2).

**Table 2:** Findings from included studies

Authors (Year)	Country of study	Data colleection Method/Participants	Sex/gender	Urban or rural Setting	Recruitment Setting	Sampling technique	Data collection instrument	Theoretical framework	Analysis	Key findings	Limitations reported by study authors
(Brown et al., 2015) [39]	Botswana	Focus group discussion was conducted for 12 adolescents aged 12–18 years	6 males and 6 females	Not reported	School	Not reported	Interview guide	Socio-ecological model	Thematic	a) Adolescents’ preference often favours unhealthy options due to taste preferences, peer influence, and the belief that such foods are more affordable. b) Consumption of traditional foods was linked to poverty, while consuming non-traditional was associated with wealth and social acceptance among peers. c) Parents generally support healthy eating for their children but admit to buying unhealthy foods to accommodate their adolescents’ preferences d) Both adolescents and parents view a normal body weight as ideal both for self-image and attractiveness in others. e) Traditional beliefs persist among some family and community members who continue to associate a larger body size with wealth and good health.	a) The study was only applicable to adolescents in urban areas. b) Potential cultural differences between the researcher and participants may have led to misinterpretations or misunderstandings of certain responses or issues.
(Bodiba, 2008) [45]	South Africa	Focus group discussion was conducted for 9 female adolescents aged 17–19 years	9 females	Not reported	University	Convenience	Topic guide	Not reported	Thematic	a) Adolescents with overweight expressed satisfaction with their body weight and some were unhappy with their body size and expressed a desire to lose weight. b) Adolescent with a normal body weight were content with their appearance and believed that gaining weight could negatively impact their chances of attracting a cute boyfriend. c) Some participants living with overweight or obesity reported experiencing limitations in participating in school activities such as sports and certain type of jobs d) Some participants also shared that their weight restricted their ability to wear the clothes they preferred, which impacted their sense of style and fashion choices. e) Some adolescents living with overweight reported experiencing discrimination due to their weight by their peers, with some females sharing that their boyfriends ended relationships with them for this reason. f) Most adolescents expressed dissatisfaction with how the media emphasizes weight loss and promotes the idea that being overweight is unacceptable. d) Society members also place strong emphasis on weight loss, reinforcing negative perceptions of being overweight	The study focused exclusively on female participants
(Dapi et al., 2007) [43]	Cameroon	In-depth interview was conducted for 15 adolescents aged 12–15 years	Not reported	Urban and rural	Not reported	Purposive	Interview guide	Grounded theory	Thematic	a) Adolescent girls from rural area preferred to be fat and not slim. Adolescents girls from poor urban areas prefer to gain weight. Those from urban rich areas prefer normal weight.	Selection bias, small sample size
(Getacher et al., 2023) [46]	Ethiopia	In-depth interview was conducted for 20 adolescents aged 10-19years	14 males and 6 females	Not reported	School	Purposive	Interview guide	Not reported	Thematic	a) Some students believed that being thin prevents weight gain, regardless of how much or what they eat. b) Parental economic status significantly influences adolescents’ dietary intake, as it determines the availability and variety of food at home. c) Peer pressure often encourages poor dietary choices. d) Exposure to media promotes unhealthy foods.	Limited to only schools
(Hendricks et al., 2022) [47]	South Africa	Group Model Building was conducted for 62 adolescents aged 16-18years	Not reported	Not reported	School	Purposive	Group Model Building	Not reported	Causal loop diagram	a) Unhealthy dietary habits among adolescents were linked to parental involvement. b) Some adolescents reported that when parents failed to meet their emotional needs, they sought attention and affection elsewhere, which often led to unhealthy eating behaviours. c) Lack of parental engagement in encouraging physical activity, monitoring eating habits, and providing nutritious meals affect their weight. d) Parents working long hours and lacking time leads to irregular mealtimes, increased snacking on unhealthy foods, and reliance on convenience meals. b) Many expressed concerns about weight stigma, noting its association with discrimination, reduced job opportunities, and lower income. d) Some adolescents reported that contraceptive use affected their appetite and contributed to weight gain. e) Adolescents preferred socializing with peers, often involving the intake of unhealthy foods f) Adolescents noted a greater burden of weight-related health problems and associated psychosocial challenges in these environments.	a) The findings of this study cannot be generalized beyond the specific time, location, and participant group involved. b) The study focussed on adolescent perceptions, but data were collected only from ages between 16–18-year-old. As such, the results may not reflect the views or experiences of younger adolescents, out-of-school youth, or those from different social or regional backgrounds. c) Additionally, differences in understanding and interpretation of the questions may have influenced participants’ responses.
(Jallow-Badjan et al., 2025) [40]	Gambia	Focus group discussion was conducted for 32 adolescents aged 10–19 years	32 females	Urban and rural	Not reported	Purposive	Interview guide	Socio-ecological model	Thematic	a) Some households advised adolescents to avoid specific types of foods, such as meat or fish. b) Consuming both lunch and dinner were perceived to maintain healthier body weight. c) Adolescents believed that overweight and obesity result from consuming foods that are high in energy density. d) Adolescents believed that parental restrictions on the consumption of unhealthy foods could help prevent excessive weight gain. e) Some adolescents also suggested that community leaders should provide safe and accessible spaces for physical activity	A limitation of the focus group discussion was that certain individuals dominated the conversation, which may have restricted input from other participants. The small sample size may have resulted in a selection bias
(Janha et al., 2021) [48]	Gambia	Focus group discussion was conducted for 40 adolescents aged 10-17years	Not reported	Rural	Community	Purposive	Focus Group Discussion guide	Not reported	Thematic	a) Many adolescents perceived mothers as the primary decision-makers for the main daily meal, with fathers playing a secondary role. b) Food-sharing practices were shaped by tradition, household size, and gendered family hierarchies. c) These practices often resulted in adolescent boys receiving more nutritious food than girls. d) Festivals and celebrations such as births and weddings were also seen to occasionally impact adolescent diets. e) Cultural practices further influenced physical activity. Music and dance were widely regarded as key motivators. f) Participants believed that frequent use of media devices negatively affected physical activity levels. g) Rural adolescents still engaged in various forms of physical activity due to environmental factors. h) Gender norms influenced physical activity opportunities, with boys’ use of recreational spaces limiting opportunities for girls.	The authors reported that some adolescents dominated the discussions, whereas others were unwilling to participate or provide more detailed responses
(Kataria et al., 2022) [49]	South Africa	Interviews were conducted for adolescents aged 15–19 years	Not reported	Urban and rural	Not reported	Purposive	Interview guide	Not reported	Thematic	a) Experiencing negative comments about their weight was reported to negatively impact adolescents’ psychological well-being leading to low self-esteem. b) Some adolescents felt that others judged them based on their body weight.	Participants differed from non-participants, making the findings less representative.
(Puoane et al., 2010) [50]	South Africa	Focus group discussion was conducted for 60 adolescents aged 10–18 years	60 females	Not reported	School	Random selection	Interview Questions	Not reported	Thematic	a) Overweight was commonly perceived by adolescents as a sign of health and beauty, while thinness was often associated with illness. b) overweight was viewed as a sign of respectability and was believed to enhance appearance, especially in clothing. c) Adolescents living with overweight often experienced social exclusion, as peers tended to isolate them, suggesting a lack of acceptance within peer groups. d) Parents who associated thinness with sickness tended to prefer their children to be overweight, which influenced adolescents’ attitudes toward body weight and health.	Limited to adolescent girls
(Seabi et al., 2021) [44]	South Africa	Focus group discussion was conducted for 31adolescents aged 14–19 years	16 males and 15 females	Rural	Community	Convenience	Interview Questions	Socia Cognitive Theory (SCT)	Thematic	a) Some adolescents associated obesity with happiness and a life free from worries or problems. b) Others held the view that individuals living with obesity are lazy. c) Having family members who are living with obesity was seen as a contributing factor to an adolescent’s weight gain. d) The use of contraceptives was also identified by some adolescents as a cause of obesity. e) Adolescents believed that unhealthy food choices also cause obesity.	a) SCT used in this study limited the findings representativeness. b) Researcher bias
(Sedibe et al., 2014) [51]	South Africa	Interviews were conducted for 22 adolescents aged 16–19 years	22 females	Rural	Not reported	Purposive	Interview guide	Not reported	Thematic	a) Some adolescents believed that traditional foods are healthy. b) majority indicated that their consumption of healthy foods largely depended on availability. c) Affordability and accessibility of healthier food options were identified as major contributing factors to obesity. d) Peer influence was also reported as a significant factor that could either support or hinder the adoption of a healthy lifestyle.	The study was only focused among adolescent girls limiting its representativeness
(Tshililo et al., 2016) [52]	South Africa	Focus group discussion was conducted for 50 adolescents aged 13-19years	50 females	Not reported	School	Purposive	Not reported	Not reported	Thematic	a) Adolescents living with overweight reported experiencing teasing from family members. b) Weight stigma from peers’ shapes adolescents’ weight control behaviours. c) Peer approval or disapproval of dietary choices was found to influence how adolescents managed their weight.	Limited to adolescent girls
(Wanjohi et al., 2025) [53]	Kenya	Focus group discussion was conducted for 102 adolescents aged 10–19 years	51 males and 51 females	Urban	Community	Purposive	Focus Group Discussion guide	Not reported	Thematic	a) Unhealthy and processed foods were often perceived as “classy” and associated with higher socioeconomic status. b) Peer pressure was identified as a key driver of unhealthy food consumption among adolescents.	a) Desirability bias b) The findings were from adolescents’ perspective therefore cannot be representative
(Voorend et al., 2013) [54]	South Africa	Interview was conducted for 58 adolescents	58 females	Urban	School	Not reported	Interview guide	Not reported	Content	a) Adolescents reported consuming the same food as their peers. b) Food choices are often shared and jointly decided by friends. c) The foods commonly purchased at school were largely unhealthy. d) Factors such as availability, price, and quality were reported to influence the selection of foods bought at school.	a) The study was dominated by some adolescents. b) Researcher bias influences the findings
(Buyinza et al., 2025) [41]	Uganda	Focus group discussion was conducted for adolescents aged 10–19 years	Not reported	Rural	School and Community	Purposive	Focus group discussion	Socio-ecological	Thematic	a) Adolescents perceived certain foods as important for satisfying hunger and providing energy. b) Food choices were based on taste, visual appeal and smell of the food c) Adolescents associated the consumption of sweet and fatty foods with enhanced beauty and greater sexual attractiveness. d) Adolescents reported adopting the eating behaviours of their peers to fit in with social groups and avoid social exclusion e) Parents’ food choices and the availability of foods at home influence adolescents’ eating behaviours. d) Cultural norms and gender-specific food taboos restricted adolescent girls from consuming certain foods such as chicken and lungfish. f) Media influenced adolescents’ eating behaviours through celebrity endorsements, television advertising, and indirectly through peers who shared media-promoted messages about certain foods. g) Adolescents reported that food choices were based on physical access f) Gender-specific cultural food taboos influenced dietary behaviours, particularly by restricting certain foods among girls.	The study was self-reported and may be influence by adolescents perception or social desirability bias
(Agedew et al., 2022) [55]	Ethiopia	Interview was conducted for 12 adolescents (10–19)	Not reported	Urban and Rural	Community	Purposive	In-depth interview	Not reported	Content analysis	a) Foods available and easily accessible at home influence adolescent food choices because they consume foods that are available and served at home. b) Family dietary habits influence adolescents’ food choices c) Adolescents reported that nutritious foods were primarily provided to young children and sick family members. d) Girls reported lower preference for some animal-source foods, indicating gender-related influences on dietary diversity.	The study does not allow for the estimation of the prevalence of different food items consumed
(Mama Chabi et al., 2022) [42]	Benin	Focus group discussion for 77 adolescents and interviews for 7 adolescents aged 15 to 19 years	77 females	Urban	School	Purposive	Focus group discussion and Interviews	Socio-ecological	Thematic	a) Adolescents’ food preferences influenced their food choices and consumption behaviours. b) Family eating habits and parental role modelling influenced adolescents’ food choices, as they often consumed the foods prepared and provided at home by their mothers. c) Adolescents reported readily adopting their peers’ food preferences, particularly regarding unhealthy dietary behaviours such as eating snacks. d) Advertisements for unhealthy foods in the media and TV motivated adolescents to consume snacks, fast foods, and pancakes.	The study did not examine gender differences in peer influences.

### Quality Assessment of Included Studies

All 17 studies scored between 8 and 10 in the CASP tool, indicating strong methodological reliability and usefulness for the purpose of this review (Table 3). Each of the studies clearly stated the research objectives and used appropriate qualitative methodologies that aligned with the study aims. All the studies had ethical approval. The study designs for all the studies were suitable and recruitment strategies and data collection methods were considered appropriate to address their respective research questions. Data analysis was described in detail in the studies and findings were clearly presented. Out of the 17 studies, eight did not address the researchers’ positionality or acknowledge potential biases that may have influenced the research process and findings.

**Table 3:** Quality assessment

Author (Year)	Was there a clear statement of the aims of the research?	Is a qualitative methodology appropriate?	Was the research design appropriate to address the aims of the research?	Was the recruitment strategy appropriate to the aims of the research?	Was the data collected in a way that addressed the research issue?	Has the relationship between researcher and participants been adequately considered?	Have ethical issues been taken into consideration?	Was the data analysis sufficiently rigorous?	Is there a clear statement of findings?	How valuable is the research?
(Brown et al., 2015) [39]	Yes	Yes	Yes	Yes	Yes	Can’t determine	Yes	Yes	Yes	Yes
(Bodiba, 2008) [45]	Yes	Yes	Yes	Yes	Yes	Yes	Yes	Yes	Yes	Yes
(Dapi et al., 2007) [43]	Yes	Yes	Yes	Yes	Yes	Yes	Yes	Yes	Yes	Yes
(Getacher et al., 2023) [46]	Yes	Yes	Yes	Yes	Yes	Yes	Yes	Yes	Yes	Yes
(Hendricks et al., 2022) [47]	Yes	Yes	Yes	Yes	Yes	Yes	Yes	Yes	Yes	Yes
(Jallow-Badjan et al., 2025) [40]	Yes	Yes	Yes	Yes	Yes	Yes	Yes	Yes	Yes	Yes
(Kataria et al., 2022) [49]	Yes	Yes	Yes	Yes	Yes	Can’t determine	Yes	Yes	Yes	Yes
(Puoane et al., 2010) [50]	Yes	Yes	Yes	Yes	Yes	Can’t determine	Yes	Yes	Yes	Yes
(Seabi et al., 2021) [44]	Yes	Yes	Yes	Yes	Yes	Yes	Yes	Yes	Yes	Yes
(Sedibe et al., 2014) [51]	Yes	Yes	Yes	Yes	Yes	No	Yes	Yes	Yes	Yes
(Tshililo et al., 2016) [52]	Yes	Yes	Yes	Yes	Yes	Yes	Yes	Yes	Yes	Yes
(Wanjohi et al., 2025) [53]	Yes	Yes	Yes	Yes	Yes	Yes	Yes	Yes	Yes	Yes
(Voorend et al., 2013) [54]	Yes	Yes	Yes	Yes	Yes	No	Yes	Yes	Yes	Yes
(Buyinza et al., 2025) [41]	Yes	Yes	Yes	Yes	Yes	No	Yes	Yes	Yes	Yes
(Janha et al., 2021) [48]	Yes	Yes	No	Yes	Yes	Yes	Yes	Yes	Yes	Yes
(Agedew et al., 2022) [55]	Yes	Yes	Yes	Yes	Yes	No	Yes	Yes	Yes	Yes
(Mama Chabi et al., 2022) [42]	Yes	Yes	Yes	Yes	Yes	No	Yes	Yes	Yes	Yes

Analysis of the findings of these studies revealed the following insights on perceived peer, family and media influences on overweight and obesity and experiences of weight stigma.

### Peer Influences

Nine studies reported that adolescents considered peers to influence their weight status [39, 41, 42, 46, 47, 51–54]. Adolescents reported that peer influences could support or hinder healthy behaviours [51]. To illustrate, it was highlighted by adolescents in Botswana that the consumption of unhealthy food is often normalized and socially accepted within peer groups [39]. Adolescents who participated in the study further explained that their peers also made mocking comments when they chose to eat healthy foods, and this discouraged them from making healthy choices. In a similar study in Uganda, adolescents reported adopting some unhealthy eating behaviours their peers to fit in with social groups and avoid social exclusion [41]. Some adolescents in Benin also reported adopting their peers’ food preferences, particularly snacks [42]. In Ethiopia, adolescents identified peer pressure as a key factor contributing to adolescent malnutrition, as peers mostly encouraged poor dietary choices and unhealthy eating behaviours [46]. Adolescents in South Africa indicated that they often consumed unhealthy foods when spending time with friends, which they believed contributed to increased weight [47]. In another study from South Africa, adolescents reported that approval or disapproval from their peers regarding dietary choices was perceived to influence how they managed their weight [52]. For instance, one adolescent reported that they and their friends replaced regular meals with snacks as a strategy to lose weight. In another study, adolescents indicated that decisions about what to eat were made collectively among friends and that these decisions tended to favour unhealthy foods [54]. Kenyan adolescents reported being drawn to unhealthy foods such as sodas, crisps, chips and French fries when their friends consumed them [53].

Adolescents living with overweight reported being excluded by peers from participating in school activities such as sports and associated these experiences with lower self-esteem [45]. Adolescents attributed experiences of exclusion by peers and teachers to their weight status [45]. These adolescents also reported that ‘societies’ emphasis on weight loss reinforced weight-related stigma. They also faced rejection from their peers and social exclusion because of their weight [50]. Weight stigma from peers was reported by adolescents living with overweight as a key factor in their efforts to manage or control their weight [52]. For example, they reported skipping meals or using weight-loss tablets in response to stigmatisation.

### Parental and Family Influences

In six of the included studies, adolescents identified parental factors (for example, role modelling) and family values as important factors related to their weight-related behaviours and body weight [39–42, 44, 46, 47, 50, 55]. In South Africa, adolescents reported consuming comfort foods and snacks when they felt their emotional needs were not met by their parents [47]. These adolescents also reported that parents did not promote a healthy lifestyle. For example, they noted limited encouragement of physical activity, limited monitoring of eating habits, and minimal nutritious meals provided at home [47]. Adolescents attributed this lack of health promotion by parents to long working hours, which they associated with irregular mealtimes, increased snacking on unhealthy foods, and reliance on convenience meals [47]. Parents tend to provide high-calorie foods for adolescents because they think adolescents prefer them [39]. Adolescents perceived their family’s economic status, level of education, and knowledge of food and nutrition as influential factors on their dietary intake [46]. Adolescents perceived the amount of money available and/or parental knowledge as important influences on the variety and quality of food available at home[41, 42, 55]. Furthermore, adolescents reported that parents who associated thinness with sickness tended to prefer their children to be overweight, and they perceived these parental beliefs as shaping their own attitudes about weight and health [50]. Weight-related stigma was not mentioned in this study, but another study found that adolescents perceived family members as influencing their weight control strategies, including the use of tablets or skipping meals to lose weight [52]. Adolescents perceived having family members living with obesity as being associated with their own weight gain, as they tended to imitate these family members’ unhealthy eating and physical inactivity [44]. In the study from Gambia, adolescents said parents could help them prevent unhealthy weight gain by placing restrictions on the consumption of unhealthy foods [40].

### Media Influences

Four studies indicated that adolescents perceived exposure to unhealthy foods in the media as being associated with their weight status [39, 41, 42, 45, 46, 48]. Adolescents acknowledged media as an important source of information and one that they relied on [39]. Media promotion of unhealthy foods was identified by adolescents in Ethiopia as a risk factor for obesity [46]. The study conducted in South Africa described a strong disconnect between self-perceived body image and media portrayals of body image, which often idealise thinness as the standard for female beauty while depicting overweight bodies as undesirable [45]. This is likely to lead to increased weight stigma (from peers for example), but weight stigma was not mentioned in the study. In the study from Gambia, adolescents reported that excessive use of media devices such as watching television reduced their engagement in physical activity, which they perceived as a contributing factor to weight gain [48]. In another study in Uganda, adolescents perceived the media as shaping their eating behaviours through celebrity endorsements, television advertising, and indirectly through peers who shared media-promoted messages about certain foods [41]. For instance, they indicated that foods advertised on television looked appetising and delicious, which they perceived as increasing their desire to consume such foods. Similarly, in Benin, adolescents reported that advertisements of unhealthy foods in media/TV motivated them to consume snacks and fast foods [42].

### Gender-Related Socio-cultural Influences

Gender-related influences on adolescents’ weight-related perceptions and behaviours were reported in ten studies. In Ethiopia, adolescent girls described avoidance or low preference for some animal-source foods [55]. Gendered dietary practices were also reported in Uganda, where cultural food taboos restricted the consumption of certain foods, particularly among girls [41]. In Cameroon, body-size preferences among females varied in urban and rural settings, with larger body sizes particularly valued among girls in rural settings [43]. Adolescent girls in South Africa described the influence of body size and appearance on body image and self-concept [45] while cultural perceptions of larger bodies as attractive, healthy, or socially desirable were also reported among girls [50]. Family and peer influences were evident in girls’ eating and physical activity behaviours [51], weight-control practices [52] and food purchasing and sharing practices within female friendships [54]. In Benin, girls described family, peer, and food-environment influences on their eating habits [42], while findings from Gambia highlighted social, cultural, household, and food-environment influences on nutrition among adolescent girls [40]. Gender norms also influenced opportunities for physical activity in rural Gambia, where boys’ use of football and recreational spaces was perceived as limiting opportunities for girls to participate in physical activity [48].

### Economic and Environmental Influences

Eight studies reported economic and environmental factors that adolescents perceived as relevant to their dietary and physical activity behaviours. In Botswana, adolescents perceived unhealthy foods as more affordable, while traditional foods were associated with poverty and non-traditional foods with wealth and social acceptance [39]. In Ethiopia, parental economic status was reported to influence dietary intake through the availability and variety of foods at home [46]. Another study in Ethiopia described the availability and accessibility of foods at home as relevant to adolescents’ food choices [55]. Similarly, in Kenya, unhealthy and processed foods were perceived by adolescents as “classy” and associated with higher socioeconomic status [53]. In South Africa, adolescents reported that the availability, affordability, and accessibility of healthier foods were relevant to their dietary behaviours [51]. Another study found that the availability, price, and quality of foods influenced foods purchased at school [54]. In Gambia, adolescents identified the need for safe and accessible spaces for physical activity [40, 48]. In Uganda, food availability at home and physical access to food were perceived by adolescents to influence their eating behaviours [41].

### Cultural Connections Between Overweight and Obesity, Health, Wealth, Beauty and Happiness Through Peer, Family and Media Influences

The following themes capture the cultural beliefs and experiences associated with overweight and obesity among adolescents in African countries. They describe how body size, food practices, the causes and management of overweight and obesity, and experiences of weight stigma are understood and interpreted within different African countries.

### Cultural Beliefs About Body Size, Health and Social Status

Ten studies (from South Africa, Botswana, Cameroon, Uganda, Ethiopia, Gambia and Kenya) reported that culturally, overweight and obesity were associated with positive attributes [39, 40, 43–46, 50, 51, 53]. Adolescents in Botswana reported that there were traditional beliefs that associate overweight with wealth and good health [39]. Another study in South Africa revealed that overweight was perceived by adolescents themselves as a sign of health and beauty, while thinness was associated with illness [50]. In South Africa, some adolescents expressed the belief that being overweight was part of their African heritage and, as a result did not perceive their weight as problematic [45]. For instance, one adolescent living with overweight reported being unconcerned about negative comments regarding her weight and expressed satisfaction with her current body size as it reflected a typical figure of an African. Adolescents in Cameroon perceived overweight as a standard of beauty, where beauty was associated with fatness and not slimness [43]. Adolescents expressed the belief that overweight was viewed positively in their communities, as it is associated with enhanced appearance, especially regarding how clothing fits [50]. Overweight and obesity were associated with happiness by adolescents in South Africa and with a life free from worries or problems [44].

### Cultural Beliefs About Food and Dietary Practices

Findings from Ethiopia indicated that some students believed thin individuals could not become overweight irrespective of dietary intake [46]. Moreover, adolescents reported a belief that consuming traditional diets was associated with poverty, while consuming high calorie and processed foods was associated with wealth [39]. Some traditional foods were associated with health however, with adolescents in one study reporting that foods such as green leafy vegetables, legumes and nuts were healthier, and that this perception encouraged them to eat healthy foods [51].

### Cultural Beliefs About the Causes and Management of Overweight and Obesity

In South Africa, adolescents shared their views that the use of contraceptives and Human Immuno-deficiency Virus (HIV) medications were associated with overweight and obesity [44, 47]. It was reported that contraceptive use affected eating habits, changes their appetite, and may contribute to weight gain [47]. Some adolescents also attributed obesity among females to the use of oral or injectable contraceptives, whereas others associated overweight and obesity with the side effects of HIV-related medications and treatment. Conversely, male adolescents reported using substances such as marijuana for weight reduction with the belief that it would also help to relieve psychological stress [44].

### Cultural Beliefs, Weight Stigma and Social Consequences

Some adolescents with thin bodies reported experiencing weight stigma, indicating that weight-related stigma can occur regardless of body size. For example, thinness was associated with illness, and being thin or slim was linked to diseases such as HIV/AIDS or Tuberculosis (TB) [50]. Moreover, weight stigma was reported more in older studies[45, 50, 52] compared to recent ones[47, 49] suggesting that stigmatisation may become less prominent over time.

Adolescents expressed concerns about the long-term implications of weight stigma, noting its association with discrimination, reduced job opportunities, and lower income [47]. They also expressed immediate implications as a pressure they felt to meet body image and shape ideals [47]. Experiencing negative comments about their weight was reported to be associated with poor psychological well-being[49] because they felt they were being judged for their weight.

## Discussion

### Main Findings

We found 17 qualitative studies from eight African countries that describe adolescents’ views on socio-cultural influences on overweight and obesity and experiences of weight stigma in African countries. Adolescents perceived their peers as influencing their dietary habits primarily by promoting consumption of unhealthy foods. Parents and other family members were perceived to influence adolescents’ eating habits and lifestyle choices directly, for example through the foods they provided, or socially through poor role modelling. They also perceived media images and promotions as reinforcing both positive and negative peer and family influences on their weight-related behaviours and perceptions. Weight stigma was reported among adolescents irrespective of their weight status.

### Comparison with the Literature

We found that adolescents value peer relationships and social belonging, and may adopt behaviours and lifestyles in common with people in their social circles. We observed this for adolescents in several African countries, but similar evidence has been observed globally in developed and developing countries [56–59]. In this review, adolescents said peers pressured them towards unhealthy food choices [60]. High consumption of unhealthy diets among adolescents can be attributed to the affordability, accessibility, and convenience of unhealthy foods [61, 62]. Furthermore, our study revealed that healthy food choices made by adolescents were often undermined by teasing or disapproval from peers. This finding corroborates previous studies, [63–65] highlighting the strength of peer influence on adolescent dietary behaviours. The normalisation of unhealthy food consumption within peer groups not only undermines efforts to promote healthy eating but also normalises negative attitudes toward healthy lifestyles. To meet peer expectations of body standards, adolescents reported using unhealthy practices such as skipping meals or replacing them with snacks to control weight. However, this is unlikely to be successful because adolescents who use unhealthy weight control strategies such as meal skipping do not experience weight loss [66]. These unhealthy behaviours also have important nutritional implications because meal skipping and inadequate food intake can contribute to undernutrition and nutrient deficiencies [67]. On the other hand, consumption of snacks that are energy-dense and nutrient-poor foods can lead to overweight and obesity [68].

The provision of high-calorie and low-nutrient foods at home, combined with limited promotion of physical activity, may create a setting where poor dietary habits are easily adopted and sustained [69]. Eating or physical behaviours of families (healthy and/or unhealthy) are likely to be adopted by adolescents who emulate behaviours of their parents [70]. Our findings align with previous study suggesting that parental practices and beliefs influence adolescent eating behaviours [71]. Eating behaviours of children and adolescents are shaped by their families’ food choices and taste preferences [72]. This is consistent with literature showing that multiple family-level variables, including socioeconomic status, education, cultural beliefs about body image, and parental role modelling contribute to adolescents’ dietary behaviours [73, 74].

Our findings highlight that adolescents may experience conflicting influences on body-size ideals. Larger bodies were clearly valued in some cultural contexts while thinner bodies were idealised in the media. Adolescents associated overweight and obesity with positive attributes such as health, beauty, happiness and wealth, whereas thinness was sometimes associated with illness [39, 40, 43]. At the same time, some adolescents reported that media idealised thinness and presented larger bodies negatively. These findings suggest that adolescents may be exposed to multiple, and sometimes conflicting messages about what constitutes a desirable body. The findings also suggest that cultural preferences for larger body sizes co-exist alongside thinner body ideals.

This coexistence may help explain the ‘stigma-paradox’ we observed where experiences of weight stigma were reported by adolescents regardless of their weight status. The included studies did not provide sufficient evidence to identify body-size thresholds above or below which stigma occurred. Instead, stigma appeared to occur when adolescents’ body weight was perceived as inconsistent with prevailing social expectations of desirable body size. Adolescents living with overweight reported teasing, exclusion and pressure to control their weight [49, 50], while some thin adolescents reported being stigmatised because thinness was associated with illness or poor health [50]. Media exposure may add to the complexity by introducing or reinforcing thinner body ideals alongside existing cultural preferences for larger bodies. Because the included studies were qualitative in nature, our findings cannot determine if media exposure is driving a transition in African body-size norms. Further research is needed to explore this and examine how competing ideals develop and interact in different African contexts. Our findings contradict studies from developed countries, where overweight and obesity are often associated with low socio-economic status, while thinness is linked to attractiveness, success, and social acceptance [75–77].

The adverse experiences associated with weight stigma observed in this study have been observed previously [78, 79]. Other studies have found that weight stigma experiences from peers are mostly in the form of teasing, exclusion, labelling, and stereotyping [24, 80]. Also, stigma from parents and family members can manifest through negative comments about adolescents’ weight status or pressure from them to lose or gain weight that conforms to their ideal body weight [81–83]. These comments can internalize negative self-perceptions and contribute to poor body image. Furthermore, parents may unknowingly reinforce stigma through their own beliefs about body size, health, or cultural standards of beauty and social status.

Our study identified gender norms as relevant to adolescents’ opportunities for physical activity, with boys reported to occupy recreational spaces such as football fields more often, potentially limiting girls’ participation. This finding is consistent with previous research among adolescent girls, which reported that gender norms and social and environmental factors were relevant to girls’ participation in physical activity [84]. These findings suggest that opportunities for physical activity may relate to both access to recreational spaces and gender-related norms surrounding their use. Moreover, our review found that body-size preferences among adolescent girls varied across social and cultural contexts, with differences in preferred body sizes reported between rural and urban settings. This is consistent with findings from a previous systematic review of women and adolescent girls in Africa, which reported variation in preferred body size according to place of residence and socioeconomic context [85]. These findings suggest that perceptions of a desirable body size among adolescent girls may not be uniform across African settings but may reflect different social and cultural expectations about body size.

Our finding that food availability, accessibility, affordability, and school and community food environments were perceived as relevant to adolescents’ dietary behaviours is consistent with evidence from high-income countries [86, 87]. The availability and accessibility of healthy foods were reported by adolescents to support healthy eating, whereas the high availability and low cost of unhealthy foods were identified as barriers [87]. Similarly, a systematic review of 36 studies from high-income countries found that greater exposure to unhealthy food outlets was generally associated with less healthy food purchasing and dietary intake among adolescents [86]. The similarity in these findings suggests that, despite differences in cultural and economic contexts, adolescents’ food choices are influenced to a great extent by the food environments in which they live.

### Implications for Interventions

The findings from this review can inform the development of interventions to improve obesity-related food consumption behaviours among adolescents by emphasising social and cultural influences. For example, interventions may be more effective if they involve families, improve parental knowledge of healthy diets, encourage healthy food provision and positive role modelling at home, and raise awareness of how cultural beliefs about gender, body size and health may encourage unhealthy eating and weight-based stereotypes [88, 89]. Peers could be empowered to promote healthy food choices and counter media pressure to consume unhealthy foods. The media could be used to build adolescents’ capability to critically evaluate unhealthy food advertising and celebrity endorsements [89].

### Strengths and Limitations of the Study

The strengths of this review include the systematic gathering and comprehensive synthesis of qualitative data to improve understanding of adolescents’ perceptions of the social and cultural influences on weight gain through peers, families and the media across eight African countries. The use of detailed and inclusive search terms ensured broad coverage of the literature to minimize the risk of missing relevant studies. Another strength is the rigorous methodological approach employed for the study selection and data extraction to enhance the reliability of the findings. All studies included were assessed for methodological quality to strengthen the reliability of the synthesis. Notwithstanding the strengths of this study, the review has several limitations. First, while socio-cultural theory provided useful guidance for understanding adolescents’ perceptions of social and cultural themes related to weight and weight-related behaviours, reliance on a single theory may mean we missed other important influences explained by alternative theories. The coding approach allowed additional economic, environmental, and gender-related factors to be identified, these were not the primary focus of the review and may not have been captured comprehensively. In addition, only English articles were included, and this may introduce selection bias such that relevant studies published in other languages were excluded.

The geographic distribution of the included studies suggests careful consideration when interpreting findings. Eight of the included studies (8 out of 17) were conducted in South Africa, and no eligible studies were identified from Northern or Central Africa. As a result, some of the themes identified in this review, particularly those relating to cultural beliefs about body size, health, beauty and social status, may reflect socio-cultural contexts in South Africa in particular, rather than shared cultural norms across Africa. It is reasonable to expect that cultural practices, social norms and ideal body size vary between African countries and as such, our findings should be interpreted as a synthesis of the available evidence from the eight included countries rather than as representative of African adolescents generally.

This review was limited to adolescents’ perceptions of socio-cultural influences on overweight/obesity and therefore did not incorporate the perspectives of parents, teachers, healthcare professionals, community leaders, or other individuals who may influence adolescents’ health behaviours. Several included studies focused exclusively on adolescent girls, while others did not report or analyse findings separately by gender, limiting our ability to compare socio-cultural influences and weight-stigma experiences between male and female adolescents.

Finally, several methodological limitations were identified across the included studies. These included small sample sizes, the restriction of some studies to adolescent girls or particular age groups, and recruitment from specific geographical or institutional settings, which may limit the transferability of the findings to other adolescent populations. Some studies reported potential selection or social desirability bias, while others identified possible researcher bias or cultural differences between researchers and participants. In some focus group discussions, a small number of participants may have dominated the conversation, limiting the contributions of others.

## Conclusion

This study aimed to describe adolescents’ views on socio-cultural influences on overweight and obesity and experiences of weight stigma in African countries, and we found that adolescents perceived peers, families, and media as important socio-cultural influences on their behaviours, weight and experiences of weight stigma. Through these influences, overweight and obesity were mostly associated with health, beauty, or wealth. Experiences of weight-related stigma were reported among adolescents regardless of their weight status. These findings suggest that socio-cultural influences should be more prominent in national strategies to promote healthy weight among adolescents. For instance, media campaigns could challenge assumptions that associate thinness with illness by portraying healthy adolescents with a variety of body sizes. Campaigns could also demonstrate that weight is multifactorial and that appearance is not a diagnosis of a health condition or an indicator of contraceptive use. Nutrition interventions could demonstrate the essential nutrient content and reproductive benefits of meats like chicken to counter gender-related food taboos. Further research is needed to understand how these social and cultural influences interact with other determinants of obesity to reduce weight stigma and promote healthy weight in African countries.

## Key References

- Jallow-Badjan, H., T.A. Apekey, and M.J. Maynard, *Factors influencing malnutrition among adolescent girls in The Gambia: a mixed-methods exploratory study.* BMC Public Health, 2025. **25**(1).
  - ○ Socio-cultural and economic factors influenced adolescent nutritional status.
- Buyinza, T., et al., *Socio-ecological factors influencing dietary behaviours among adolescents and young adults in rural Eastern Uganda: A qualitative study.* PLOS One, 2025. **20**(12): p. e0337797.
  - ○ Peer, family, and environmental factors influenced adolescent dietary behaviours.
- Wanjohi, M.N., et al., *Drivers and solutions to unhealthy food consumption by adolescents in urban slums in Kenya: A qualitative participatory study.* Public Health Nutrition, 2025: p. 1–37.
  - ○ Unhealthy food consumption among adolescents were driven by affordability, availability, and social environment.
- Getacher, L., B. Wondafrash Ademe, and T. Belachew, *Lived Experience and Perceptions of Adolescents on Prevention*,* Causes and Consequences of Double Burden of Malnutrition in Debre Berhan City*,* Ethiopia: A Qualitative Study.* International Journal of General Medicine, 2023. **Volume 16**: p. 337–356.
  - ○ Pressure from peers encourages adolescents to make poor dietary choices.
- Wang, C., et al., *Assessing Weight Stigma Interventions: A Systematic Review of Randomized Controlled Trials.* Current Obesity Reports, 2025. **14**(1).
  - ○ Weight stigma interventions showed potential effectiveness in improving health-related outcomes.
- Lawrence, S.E., et al., *Family-based weight stigma and psychosocial health: A multinational comparison.* Obesity, 2023. **31**(6): p. 1666–1677.
  - ○ Family-based weight stigma was linked to poorer psychosocial health outcomes among adolescents.
- Hemmingsson, E., et al., *The social origins of obesity within and across generations.* Obesity Reviews, 2023. **24**(1): p. e13514
  - ○ Weight gain is influenced by intergenerational and social environmental factors.
- Anastasiadou, D., et al., *Experienced and internalized weight stigma among Spanish adolescents.* BMC Public Health, 2024. **24**(1).
  - ○ Internalised weight stigma was associated with worse psychological and health outcomes in adolescents.

## Supplementary Information

Below is the link to the electronic supplementary material.

Supplementary Material 1 (DOCX 24.8 KB)

## Data Availability

No datasets were generated or analysed during the current study.

## Abbreviations

PRISMA: ScR-Preferred Reporting Items for Systematic and Meta Analysis Scoping Review
CASP: Critical Appraisal Skills Programme
TB: Tuberculosis
HIV: Human Immuno-deficiency Virus
