# Supplementary material for: Adolescents’ perspectives on socio-cultural influences on overweight and obesity in African countries: a systematic scoping review of qualitative studies
Source: Curr Obes Rep. 2026 Sep 30;15(1):89. doi: 10.1007/s13679-026-00767-8 (PMC13627244; doi:10.1007/s13679-026-00767-8)
Supplement: Supplementary file 1 [file 13679_2026_767_MOESM1_ESM.docx]

**Appendix 1: Coding framework for qualitative synthesis**

| **Theme** | **Code** | **Definition** |
| --- | --- | --- |
| **Peer influences** | Peer food choices | Adolescents’ descriptions of peers shaping or contributing to the types of foods they choose or consume. |
|  | Peer pressure to consume unhealthy foods | Encouragement, expectations, or pressure from peers to consume unhealthy foods or sugar-sweetened beverages. |
|  | Peer eating norms | Unhealthy or healthy eating behaviours perceived as normal or socially acceptable within peer groups. |
|  | Peer role modelling | Adolescents copying or adopting eating, physical activity, or weight-related behaviours observed among peers. |
|  | Peer approval or disapproval | Positive or negative responses from peers to adolescents’ food choices, body size, or weight-management behaviours. |
|  | Peer social acceptance | Perceptions that particular body sizes or weight-related behaviours contribute to fitting in, belonging, or acceptance within peer groups. |
|  | Peer physical activity support | Encouragement or participation by peers in physical activity, sport, or other active behaviours. |
| **Family influences** | Family food provision | The types, quantity, or quality of foods provided or made available by parents or family members. |
|  | Parental food control | Parental rules, restrictions, monitoring, or control over adolescents’ food consumption. |
|  | Family eating practices | Household routines and practices relating to meals, snacking, food preparation, or food consumption. |
|  | Family role modelling | Adolescents observing and imitating family members’ eating, physical activity, sedentary, or other weight-related behaviours. |
|  | Parental health promotion | Parental encouragement or support for healthy eating, physical activity, or other healthy behaviours. |
|  | Parental knowledge | Adolescents’ perceptions of parents’ knowledge or understanding of healthy eating, physical activity, or healthy weight. |
|  | Family beliefs about body size | Family beliefs about what constitutes a healthy, attractive, desirable, or undesirable body size. |
|  | Family weight-management pressure | Family comments, expectations, or actions encouraging adolescents to gain, lose, or control their weight. |
| **Media influences** | Food advertising | Exposure to advertisements or promotions for foods and beverages through television, social media, or other media. |
|  | Media-induced food desire | Adolescents’ descriptions of advertised or promoted foods as appealing, appetising, or desirable. |
|  | Celebrity endorsement | Promotion of foods, beverages, body ideals, or behaviours by celebrities or other prominent media figures. |
|  | Peer sharing of media messages | Media-promoted food or weight-related messages reaching adolescents through their peers. |
|  | Media body ideals | Media representations or messages about desirable, attractive, healthy, thin, or larger body sizes. |
|  | Media-related sedentary behaviour | Screen or media use associated with prolonged sitting, reduced physical activity, or other sedentary behaviours. |
|  | Health information through media | Media messages perceived as encouraging healthy eating, physical activity, or weight management. |
| **Cultural beliefs and norms** | Larger body size as healthy | Cultural perceptions associating larger body size with health, strength, or wellbeing. |
|  | Larger body size as attractive | Cultural perceptions associating larger body size with beauty, attractiveness, or desirable appearance. |
|  | Larger body size as wealth/status | Associations between larger body size and wealth, prosperity, happiness, or higher social status. |
|  | Thinness as illness | Beliefs that a thin body indicates sickness, poor health, poverty, or inadequate care. |
|  | Cultural expectations of body size | Socially shared expectations about the body size considered appropriate or desirable. |
|  | Cultural food beliefs and practices | Cultural meanings, values, traditions, or practices relating to particular foods and eating behaviours. |
|  | Gendered body expectations | Different cultural expectations regarding body size, food, appearance, or weight for adolescent girls and boys. |
| **Weight stigma** | Weight-related teasing | Mocking, jokes, name-calling, or negative comments directed at adolescents because of their body weight or size. |
|  | Weight-based social exclusion | Exclusion from peer groups, school activities, sport, or other social activities because of body size. |
|  | Negative body-size judgement | Negative evaluations, stereotypes, or assumptions made about adolescents because of their weight or body size. |
|  | Stigma against larger body size | Stigmatising experiences specifically reported by adolescents living with overweight or obesity. |
|  | Stigma against thinness | Negative comments, judgement, or treatment directed towards thin adolescents, including associations between thinness and illness. |
|  | Weight-control response to stigma | Attempts to lose, gain, or manage weight following weight-related comments, teasing, pressure, or stigma. |
|  | Psychological response to stigma | Emotional or psychological experiences associated with weight stigma, including embarrassment, reduced self-esteem, distress, or dissatisfaction. |
| Other contextual influences | Food affordability | The cost of food affecting adolescents’ access to healthy or unhealthy food choices. |
|  | Food availability and accessibility | Availability or ease of obtaining foods within home, school, or community environments. |
|  | School food environment | Food availability, food purchasing, or eating practices occurring within or around schools. |
|  | Physical activity environment | Availability or accessibility of spaces, facilities, opportunities, or resources for physical activity. |
|  | School and community influences | Institutional or community conditions relevant to adolescents’ eating, physical activity, or weight-related experiences. |
